# Symposium on Tuberculosis

**Published:** 1916-06

**Authors:** 


### Symposium on Tuberculosis

(1.) Under existing conditions, especially the impossibility of altogether removing foci of infection, do you consider tuberculous infection to depend rather on the virulence of the organisms or on the susceptibility of the patient? (2.) Aside from climatic and hygienic methods, do you advocate any medical treatment as having any direct action on the disease—disregarding symptomatic treatment for complications and for temporary relief? (3.) What is your opinion as to the therapeutic action of germ derivatives?

---

### Vincent Y. Bowditch, M. D., Boston, Mass.

1. It would seem to me impossible to answer this question with any degree of certainty, for the two conditions are so essential in differing degrees in all cases of tuberculosis. We certainly see cases of disease coming at times like a bolt from a clear sky in people who up to the time of its appearance have been the "picture of health." Under such conditions, the virulence of the germ would **seem** to be largely the determining factor of its appearance, and yet we have at the same time no positive proof that it is not due to some special susceptibility to the disease in one who has appeared to be well. My **impression** is, and I cannot say more than this, that the weight of evidence would be in favor of the susceptibility of the subject rather than the virulence of the germ itself as the determining factor of its appearance in the majority of cases. As I have said, however, I cannot see how this can be positively proven.

2. Apart from hygienic and climatic methods, I know of no medical treatment in which I place any faith other than the use of tuberculin in the carefully selected cases which are so situated that they can be carefully watched. From having taken an ultra-conservative position in former years against its use, I have finally in later years come to the conclusion that certain cases (not all) which under ordinary methods were gradually growing worse or were at an apparent standstill, have been greatly benefitted after the continued use of tuberculin in very small, gradually increasing, doses. As to which cases are likely to receive the greatest benefit, it is impossible to lay down any definite rule, it being a question of experimentation, more or less, in each case. As a rule, I believe patients who show temperature should not be treated in this way. I have the strong impression, too, that cases who have been treated successfully in this way are less liable to relapse than those who have not had it; this opinion being held in former years also by the late

Dr. Trudeau, who, while conservative in its use, believed it was of benefit in many cases.

3. If by this question is meant the therapeutic action of tuberculin, I have supposed that its favorable action was due to a stimulation of the blood supply to the affected part, thus favoring a condition of healing and later cicatrization. This would seem to be at least a plausible theory.

---

**Leroy S. Peters, Albuquerque, N. M.**

In the light of present ideas on infection and immunity in tuberculosis, it seems to me that the susceptibility of the patient is far more important than the virulence of the organism, although, of course, we might say that that might play a minor role. The question of infection in tuberculosis no doubt depends upon the immunity conferred by repeated inoculations in infancy or early childhood, and if the proper amount of immunity is established that patient passes through life with only tuberculous infection and not tuberculous disease. These new thories have been advanced by German investigators for a number of years and are now beginning to cause much discussion in American literature.

The medical treatment of tuberculosis has absolutely no direct action on the disease, inasmuch as today we recognize no specific. However, I believe that the hypodermatic injection of such tonics as iron arsenite and strychnine and any other medication that may tend to build up the general condition is indirectly of benefit. The treatment of complications and drugs for temporary relief must of neccessity have their place.

As for the therapeutic action of germ derivatives I believe as yet we have none that is of especial value. Harking back again to the immunity produced in early infancy and childhood, the tuberculin or serum to be of value must of necessity contain the living tubercle bacilli, and I believe that the tendency of prevention in tuberculosis lies along this line. When we consider the pathology of the disease and the biology of the tubercle bacillus the question of a specific seems almost impossible, and to make any headway in the eradication of this disease it will be necessary to fight it from the standpoint of prevention.

---

**Dr. Karl von Ruck, Asheville, N. C.**

In the present state of our knowledge, concerning the mechanism of any bacillary infection, it is impossible to separate the roles played by the virulence of the infecting bacteria, and by the susceptibility of the infected organism. Both factors are relative. The virulence of an infection may be increased by great numbers of the invasion; also by a very direct and close infection (from person to person) so that the bacteria, discharged from the host-organism, cannot be exposed to weakening influences before they are taken up by the newly infected organism.

These conditions would tend to overcome a resistance which is sufficient under usual conditions to withstand an ordinary infection. On the other hand, the resistance of the organism may be lowered by other diseases (influenza, typhoid fever, measles, whooping cough, etc.) to such a degree that a tubercle bacillus infection, which hitherto has been held latent, now becomes activated, or that a comparatively slight new infection will suffice to produce disease.

The only medicinal treatment that can possibly have any direct action upon tuberculous disease, is that with preparations derived from tubercle bacilli. Neither drugs with general (tonics, etc.) action, nor the germicides can influence the disease directly; in short, there is at present no specific chemical known which would influence tuberculosis in the same manner in which mercury and arsenic influence lues, or quinine malaria. The only direct influence is possible from specific etiological treatment, and this I advocate with certain restrictions very decidedly. Only purely tuberculous, that is to say, uncomplicated cases of the disease can be expected to respond promptly to specific treatment; in all cases with associated infections or with much destruction of tissue, the concomitant conditions tend to limit the usefulness of the specific treatment.

The action of germ derivatives is that of stimulating the tuberculous organism to produce specific antibodies against the tubercle bacillus, which may destroy the virulence and viability of the bacilli and also neutralize the toxins which reach the circulation, resulting from breaking-down tuberculous tissues.

Under proper conditions, the action of a suitable derivative of the tubercle bacillus is promptly curative; under other conditions it can only arrest the tuberculous process, while associated complicating conditions and disease processes must be treated by other means. With a careful selection of cases, and under a proper and individualizing use of tubercle bacillus preparations, the clinical results are very much better than are those obtained by the general methods alone; they are also more lasting and less prone to be followed by recurrences of the disease.

**Dr. Edward R. Baldwin, The Trudeau School of Tuberculosis, Saranac Lake, N. Y.**

Under question (1) as to the relative importance of the organism or the susceptibility of the patient, I would say that it seems to me that the question is rather undecided as yet. At the same time there are so many possible answers that it makes it difficult to say which is the important factor in any given case. In some instances no doubt virulence of the bacillus has much to do with the fact that the patient does not resist. In these cases, however, we do not know the dosage nor the place of primary infection and how disseminated the disease may have become in the first moment of invasion. I believe that all human beings are susceptible to the bacillus when it is planted in the right place. Very much, though, has to do with keeping off the infection in the general health of the individual. Doubtless, susceptibility is another name for conditions favoring infection including virulence of the bacillus, dosage, points of infection, external conditions of life, work, food, etc. As to the actual susceptibility of the tissues to the invasion of the bacillus we know little. There are certain racial and other differences which make for an acute form of the disease as contrasted with the more chronic form. Doubtless this is a matter of tissue resistance rather than general resistance. The secret of these differences is as yet unsolved.

Under the head of (2 and 3), aside from climatic and hygienic methods I do not feel that we have any specific treatment except tuberculin in some form. I advocate tuberculin to a very moderate extent in patients who have reached a stationary point in their improvement, have no acute symptoms, i.e. no fever, but do not seem to overcome the tendency to slight relapses from time to time. I believe that such patients must be in the best of nutritive condition in order to obtain benefit from tuberculin. Certain forms of tuberculosis, notably that of the skin and glands, which are ordinarily called surgical tuberculoses, are quite amenable to specific treatment in the form of tuberculin and very excellent results are obtained in these cases. In general, however, I believe that tuberculin treatment is too often used without discretion and discrimination as to the type of disease. A certain amount of familiarity with clinical tuberculosis and knowledge of the principles of vaccine therapy is a prerequisite for successful tuberculin treatment.

---

**Dr. Lawrason Brown, Saranac Lake, N. Y.**

I believe that tuberculous infection does depend in part upon the virulence of the organism. I mean by this that exposure of tubercle bacilli to light gradually weakens them. If anyone inhales such tubercle bacilli it is possible that they may have a slow or more chronic type of disease, though this can well be questioned because of the fact that if all the tubercle bacilli are not killed their progeny may quickly regain their usual virulence. The susceptibility of the patient is, I believe, an important factor, particularly later in life when many persons have been infected and probably only develop clinical disease when their resistance is greatly reduced. I know of no medical treatment that has been proved scientifically to have any direct action on the disease, disregarding symptomatic treatment. The vast majority of patients who are treated with derivatives of the tubercle bacillus derive, in my experience, little benefit. A few are wonderfully helped. I still feel that we have not exhausted all the possibilities of so-called specific therapy, but that we may yet by various procedures be able to determine what form of antigen certain patients may require, and use them in these cases with benefit.

---

**George Thomas Palmer, M. D., President of the Illinois State Association for the Prevention of Tuberculosis; Director of the Springfield Open Air Colony; Medical Director of the Springfield Tuberculosis Dispensary, Springfield, Illinois.**

For purposes of practical application I have become accustomed to differentiate rather sharply between tuberculous infection and the development of tuberculous disease, assuming that tuberculous infection often occurs without the slightest evidence or suspicion of it on the part of the individual affected. The infection may be so remote from the development of the disease that the time of infection may never be determined.

In fact, we have come to believe that thousands of individuals go through life "infected" with tubercle bacilli who never have the slightest clinical history of tuberculosis.

The important point there, is the development of tuberculous disease and in this I am impressed that the susceptibility, or the reduced resistence of the individual is an infinitely greater factor than the virulence of the organism.

Assuming that a very large percentage of people carry dormant tubercle bacilli for years or throughout life, there is more than a smattering of truth in the assertion that "tuberculosis is quite as much a disease of nutrition as an infection."

In my opinion, the treatment of tuberculosis consists of the so-called "hygienic measures" in which climate is a factor of less importance than rest, fresh air, nourishment or rigid discipline with careful medical supervision. Aside from meeting complications and symptoms, I have never found a medicinal agent to have any material effect upon the course of the disease.

I am convinced, however, that tuberculin has a distinct place in the treatment of cases of tuberculosis in which there is not evidence of disease activity,—elevated temperature, tendency to hemorrhage, marked digestive disturbance, greatly accelerated heart action or other evidence of toxemia. The indiscriminate and injudicious use of tuberculin,—which is far more wide-spread than we have been accustomed to suppose,—has done an infinite amount of harm.

Reports of clinical experience, even when accompanied by apparently painstaking laboratory investigation, are at times painfully conflicting and the evidence presented in the case of germ derivatives in the treatment of tuberculosis is open to this criticism. A careful and unprejudiced summary of the entire available literature of specific therapy in tuberculosis will lead us to no very definite conclusion. The final decision in the matter cannot be given and we must rely for the time being upon the opinion of those whose clinical experience is sufficiently large and sufficiently varied in character to be convincing.

In my sanatorium work, where the patient is rigidly controlled and surounded by ideal conditions, I cannot convince myself that tuberculin or other "germ derivatives" are especially effective. I have not been able to show that those patients receiving tuberculin do much better than those who do not receive it.

In office practice, dealing largely with ambulatory and non-active cases, chiefly among persons having good home conditions, the results are often so definite that they cannot be ignored, nor can the improvement be entirely credited to the regulation of the lives of the patients.

In dispensary practice, however, where the home conditions are uniformly bad and regulation of the patient most unsatisfactory, the results obtained from the judicious use of tuberculin in carefully selected cases, stand out most convincingly. Here there can be no question whatever as to which of several therapeutic agencies shall be credited for the benefits obtained. In my opinion, the dispensary is the place to study the real results of specific treatment and I believe that dispensary experience will justify the continued use of tuberculins and other "germ derivatives" provided the cases are properly selected and provided tuberculin is not employed as a matter of "routine." Tuberculin treatment will not succeed as "routine treatment" for unselected groups of patients.

---

**Dr. Henry Barton Jacobs, The National Association for the Study and Prevention of Tuberculosis, Baltimore.**

In answering the questions propounded, I do so with a great deal of hesitancy because of the doubt which still must exist in the minds of all as to our complete knowledge of the subject.

It would seem from the fact that tuberculosis is found chiefly among the poorer people of a city or a country and where the hygienic conditions are poorest and where intelligence is least, and the quality of food poorest and work the hardest, that the susceptibility of the patient is of greater moment than the virulence of the organism. This view, too, is supported by the fact that so many people when placed in better surroundings and more favorable conditions of hygiene and food recover. In other words, their resistence overcomes the virulence of the organism, so that it was susceptibility in the original instance which caused them to become victims of the disease.

In answer to your second question, I should say that so far as my experience goes, the only treatment yet proved of value is that which may be embraced under your definition of climatic and hygienic methods, although by the word climatic I do not mean there is great specific value in particular climates. A free open-air life in any locality under proper supervision and in hygienic surroundings with sufficient food and rest and exercise, if that be indicated, seems to be the form of treatment which secures the most favorable results. In certain carefully selected cases the production of artificial pneumothorax seems to be a favorable adjunct to the hygienic treatment.

In regard to your third question of the therapeutic action of germ derivatives, I think there can be no question but that tuberculin in a small group of cases not only hastens recovery, but insures more permanent results.

---

**L. B. McBrayer, M. D., Supt. and Chief of Bureau, The North Carolina Sanatorium for the Treatment of Tuberculosis, Bureau of Tuberculosis, State Board of Health, Sanatorium, N. C.**

I consider the removing of the foci of infection very important, in fact, all important in the prevention of the spread of the disease. I do not regard it an impossibility, but think the focus of infection should be isolated in the home if it cannot be removed to a sanatorium. Certainly nonvirulent tubercle bacilli will not produce tuberculosis. I do believe that long continued exposure to the ordinary forms of the tubercle bacillus will produce infection, in old as well as young. We know of no specific medical treatment of any value. Germ derivatives have not proved to be of any value so far as we can discern.

---

**Dr. Charles L. Minor, Asheville, N. C.**

Whether tuberculous infection depends rather on the virulence of the organisms or on the susceptibility of the patient, is a question that cannot be accurately and scientifically answered, and of which at best I can but give my general impressions.

Personally I think the susceptibility and low vitality of the patient is a much more important element than the virulence of the organisms infecting, and that if we could keep up the vitality of our submerged tenth, who are the chief victims of this disease, by good feeding, proper living, better habits, better tenements, better hours of work, better habits of recreation, we would see a great decrease in tuberculosis.

Moreover, we cannot control the virulence of the organisms, so it is useless to worry on this side of the question, and we should direct all our attention to the patient. As I have again and again insisted, after all the tuberculosis problem is more of a sociologic than medical problem, and is to be solved more by the philanthropists and the hygienists than by the practicing physician.

You ask secondly, if aside from climatic and hygienic methods I advocate any medical treatment as having any direct action on the disease, disregarding symptomatic treatment for complications and for temporary relief? What is my opinion as to the therapeutic action of germ derivatives? I suppose by this question you would ask whether I believe there is as yet any specific treatment for tuberculosis. I do not. There are a certain number of cases in which tuberculin works remarkably and beneficially, but their number is small and it is impossible to tell beforehand in what individual case Tuberculin would be suitable and beneficial. If we could tell this, if our indications for its use were sharp, accurate and scientific, it would be a much more useful and wonderful remedy than it is. As it is, the majority of special workers in this line, I think, (certainly in this country and even in Germany, where there are many enthusiasts over Tuberculin) still look upon it with considerable doubt; and while using it in selected cases, have not the great hopes for its use that one time we had.

---

**Mazyck P. Ravenel, M. D., University of Missouri, Columbia, Mo.**

In reply to your questions, I beg to make the following statements, although it is very hard to give categorical answers.

1. The disease resulting from the infection of a given individual with any organism is the resultant of the virulence of the organism on the one hand, and the resistance, or susceptibility, of the patient on the other. The dose is also a most important factor, especially in tuberculosis. I take your question, however, as an attempt to determine the relative value of practical measures for prevention. With this in view, my opinion is that the susceptibility of the individual plays a more important part than the virulence of the organism, consequently we can do much practical good by building up the resistance or immunity of the individual.

2. I do not advocate any medical treatment as having any direct action on tuberculosis, believing that no drug has been discovered which has the slightest curative or specific effect.

3. The therapeutic action of germ derivatives, by which I understand the tuberculins and vaccines, comes nearer to giving a specific action than anything known. I believe that they should be used in all cases where there is no contrary indication.

---

**F. M. Pottenger, A. M., M. D., LL.D., Medical Director, The Pottenger Sanatorium, for Diseases of the Lungs and Throat, Monrovia, California.**

Practically every child begins to come in contact with tubercle bacilli in one way or another soon after birth. If the bacilli are few in number the probabilities are that they are destroyed by the natural defenses of the child. During their destruction the cells gradually acquire the property of producing a specific defense against the tubercle bacillus and its products; so, after a short time, the child probably has both a natural and specific defense against the tubercle bacillus.

There is no doubt that constitutional susceptibility does play a part in infection, but, as yet, we have no way of even guessing at the nature of this susceptibility. The question of local susceptibility is one that is difficult to answer. Prior to the age of puberty, any tissues of the body may be involved; but, after this, pulmonary infection is far more common. I believe that this depends upon the anatomical and developmental peculiarities of the apex of the lung.

Everything that we do in the cure and treatment of tuberculosis is indirect. We have no definite method of attacking the disease that compares in any way with antitoxin in diphtheria; but we are left to indirect measures. These may be put in two general classes; first, the specific products of the tubercle bacillus, which either come from the focus of infection in the diseased area, or may be introduced artificially from without. Without these products a specific defense cannot be maintained or increased, and this specific defense is essential to the cure of the infection. This specific defense manifests itself as a reaction between the tubercle bacillus and its products, and the body cells. As a result of this action there is produced, in and near the focus of infection, a reaction which is accompanied by hyperaemia and followed by increased fibrosis. Not only are the specific products of the tubercle bacillus essential to cure, but, it is equally essential that the functional capacity of the body cells be such that when stimulated they are able to react in such a way as to heighten the specific defense.

There is nothing specific in hygienic and sanitary methods; nothing specific in fresh air, food, or properly adapted rest and exercise. Neither is there anything specific in tonic measures, whether they be medicinal or physical; but, each one of these measures has an influence in improving the functional capacity of the body cells and, to the extent that they do this, do they add to the fighting chances of the patient.

It is important that the medical profession, and laymen as well, realize that open air is not a cure for tuberculosis, and fix definitely in their minds the action that this remedy has. A suitable tonic, if it is sufficient to raise the balance of resistance in favor of the individual, will aid in the cure of tuberculosis. Sometimes, in early cases, simply a matter of changing vocation, or a few weeks rest from business worries and cares, will turn the tide in favor of the patient just as effectually as the usual open air method in another case. Improvement in the mental attitude of the patient will also often improve the functional capacity of the cells to such an extent that healing may result. So, if I were to make a statement regarding the value of these remedies, I would say that any measure, or any combination of measures, whether it be fresh air and food; or some tonic and food; or tonic and fresh air; or suitable baths and rest; or the proper mental attitude; or whether it be the application of measures which will relieve existing toxemia or complications, produces a favorable result to the extent that it is able to improve the general functionating capacity of the body cells.

From this you can draw the conclusion that any therapeutic measure which will raise the functionating capacity in an individual organ, or of the body cells as a whole, is of value in the treatment of tuberculosis. None of them are cures, but all of them may be aids.

The specific derivatives from the tubercle bacillus, the same as fresh air, good food, psychotherapy, and other tonic measures, are of value in proportion to the manner in which they are applied. I consider the derivatives of the tubercle bacillus among my best aids; and, the more experience I have in their use, the more I feel confident of their value. The great misunderstanding which arises in regard to the use of the derivatives of the tubercle bacillus in therapy, is based on a misconception of what they are and what they are expected to do; I might say a failure on the part of the men who use them in not studying them carefully and learning to apply them as they should be applied. These products should not be considered as absolute specifics. They are not able to produce a reaction within the human body that is sufficient to wholly immunize against the tubercle bacillus; but, if they are taken for their true value, they are extremely useful. Each product will produce defensive properties on the part of the body cells against those particular products of the tubercle bacillus which it, itself, contains. If it were possible to give to the body cells the entire tubercle bacillus in solution, or in a manner that it could utilize it in exactly the same proportion that it is found in the bacillus itself, I do not doubt that we could come nearer obtaining a specific result; but this we have not been able to do, so far, successfully. Whether or not the bacillus produces a complete immunity we must not lose sight of the fact that any preparation made from the tubercle bacillus will produce a focal reaction; and, in producing a focal reaction, it will cause a hyperaemia about the focus of infection, which stimulates the local cells to increased formation of fibrous tissue and hence favors healing.

---

### J. P. Crozier Griffith, M. D., Philadelphia

It goes, of course, without saying that without the conveyance of an infection by the tubercle bacillus there can be no tuberculosis. Consequently every effort must be made to prevent such infection; and I believe that it can be accomplished in very many instances. On the other hand, it is certainly equally true that although many subjects will develop tuberculosis if tubercle bacilli reach them, the majority, perhaps, need this constitutional or local susceptibility. The local susceptibility may be, of course, only a temporary one, dependent upon some local irritation, as of the mucous membrane in some portion of the body. Consequently in the way of prophylactic treatment all measures possible must be taken promptly to relieve such irritation as soon as discovered. Apart from this I believe the chief treatment must be hygienic and sanitary. I have very little confidence indeed in the employment of any drugs, and I doubt the value of any specific derivatives from the bacillus, at least at the present time. Possibly in the future some such prophylactic measure may be developed.

---

Note by the Editor: We wish to express our gratitude to the gentlemen who have so kindly responded to the invitation to take part in this symposium. It will be noted that there is substantial agreement as to the importance of susceptibility though, since tuberculosis obviously depends upon infection with the tubercle bacillus, it is difficult to word either a question or an answer so as to compare the two factors involved. In this connection, we may reiterate a personal belief that tuberculosis is the only specific infectious disease which is at all common in this country, in which the general health and resistance of the patient (aside from specific immunity) has any marked influence on the question of development or non-development of the disease after potentially efficient exposure. This statement will probably be received with incredulity but, before expressing disapproval, check off the various infections, one by one, and record your opinion as to each. We are inclined to think that you will concede the contention as to the exanthemata and the venereal diseases, as to typhoid if you consider actual experience and not a priori impressions and that, in the end, you will hesitate only regarding diphtheria and pneumonia. As to the former, the relative importance of local lesions as compared with general health must be granted as a qualification, but there is an undecided problem as to the existence of specific immunity, awaiting further experience with the Schick test and whatever modifications it may eventually undergo. As to pneumonia, it should be remembered that the definition, from the practical standpoint of statistics, is not yet based strictly on an infection with a definite micro-organism, but that it involves location and nature of pathologic process. In the case of tuberculosis there is, **apparently** a very marked difference between the chances of development, according to general health. Very rarely does one encounter tuberculosis in a person who is or has recently been in good general health, according to ordinary superficial standards—and we use this expression so as to excuse the real absurdity of speaking of a tuberculous person as being in good general health. On the other hand, we are inclined to believe that many physicians diagnose as incipient tuberculosis what is merely anaemia, auto-intoxication, or some transient interference with nutrition, not to mention the false diagnosis of some really serious disease of an entirely different nature. Yet, it may be that, for tuberculosis as for other infections, the general health in a crude sense, has very little to do with the development of the disease, the apparent, pre-tubercular, failure of the general health, being really the result of an antecedent infection which has been more or less strictly latent.

It will be noted that all the authorities who have contributed their opinions, express themselves negatively as to the real therapeutic value of any medicinal agent in the ordinary sense. So general an agreement is highly important although, of course, somewhat disappointing. It may be well to confess that this concurrence of opinion is especially valuable to the editor who, without in any sense considering any single remedy as curative, has relied to some degree upon antiseptic chemicals and those supplying certain more or less putative deficiencies in the metabolism of the tuberculous, and especially upon certain animal extracts.

The opinions as to derivatives of the tubercle bacillus differ considerably. But it is significant that they are all negative so far as the indiscriminate, inexpert, and uncritically optimistic use of these substances is concerned.

It will be noted that Dr. McBrayer speaks hopefully of the possibility of control of foci of infection. We have elsewhere expressed the opinion that, since various European countries practically stamped out leprosy by drastic measures of segregation and crude but efficient means of disinfection—largely by fire—many centuries ago, analogous measures might come quite as close to extinguishing tuberculosis today.

In propounding the questions for discussion, it seemed best to exclude the details of hygienic and sanitary methods but, especially as the County Hospital for Tuberculosis has been made a shiboleth in this state, there is one point which ought to be definitely settled. Can satisfactory preventive and arresting "cures" be conducted by each county, or other local government, within its own confines or conveniently near, or are certain climatic factors, such as altitude, relative humidity, amount of sunshine, rainfall, wind movement, average and extremes of temperature, etc., of vital importance as has been held in the past? This is no academic question but one of the highest economic, humanitarian and even in a sense, political importance. If specific climatic differences are of no importance in preventing and curing tuberculosis, the present propaganda for the establishment of county hospitals, should be supported by the united medical profession, even to the extent of making them political issues. If any one or more of the items that go to make climate are of importance in the prevention and cure of tuberculosis, it is plainly both an economic and a humanitarian error to urge the county hospital or any other analogous local philanthropic unit, unless for such localities as happen to be favorably situated. It is scarcely necessary to point out that the decision that certain climatic conditions are of importance in controlling tuberculosis, immediately removes the general problem both from the county, or city, and from the state and renders national control necessary.

We would consider it a favor if the participants in the present discussion would further express themselves upon these outgrowths from it, and we would also welcome expressions of opinion from others.

---

**Cold Abscess of Tongue.** Venot and Arnould, Gaz. Hebd. des Sciences Med. de Bordeaux, Dec. 12, 1915. The patient was a man of 50 with laryngeal and pulmonary tuberculosis. The tumor was first noted in May and had enlarged to the size of a hen's egg. There were several small lymph nodes on the same side of the neck and one had suppurated on the opposite jaw. On evacuation, the pus showed tubercle bacilli but no growth was obtained from cultures. Several punctures were made followed by injection of iodoform in ether, the tumor now being of the size of a small nut, sclerotic and probably cured.

---

**Localized Tetanus.** Ch. Monod, Acad. de Med., Nov. 23, 1915, reports a case with spasms of the upper limb, after healing. Large doses of chloral and injections of 10 C.C. of 25% solution of magnesium sulphate, 32 injections in 13 days, were employed. The author states that the symptoms correspond with the description of Follin except that the latter holds that such cases are as severe as general tetanus—implying recovery in the case mentioned.

---

**Antityphoid Vaccination in the Course of an Epidemic.** Liabot, Jour. des Prat., Dec. 18, 1915, was called to see a man of 54. He also found his three daughters at the beginning of typhoid. Other cases developed in the neighborhood and he vaccinated the whole community except those in whom typhoid had actually developed, young infants and the very aged. No further cases occurred and no mention is made of serious reactions.

